# Monoclonal enolase-1 blocking antibody ameliorates pulmonary inflammation and fibrosis

**DOI:** 10.1186/s12931-023-02583-3

**Published:** 2023-11-14

**Authors:** Wei-Ching Huang, Chi-Fen Chuang, Yung-Tsang Huang, I-Che Chung, Mao-Lin Chen, Tung-Yueh Chuang, Xiu-Li Yang, Yu-Yau Chou, Chih-Hsin Liu, Nai-Yu Chen, Chun-Jen Chen, Ta-Tung Yuan

**Affiliations:** 1 HuniLife Biotechnology Inc, Taipei, Taiwan; 2 Department of Biochemical Science and Technology, College of Life Science, National Taiwan University, Taipei, Taiwan; 3 Department of Research and Development, HuniLife Biotechnology Inc, Rm. 1, 6F., No. 308, Sec. 1, Neihu Rd., Neihu Dist, 114 Taipei City, Taiwan

**Keywords:** Enolase-1, Antibody, Plasmin, Migration, Fibroblasts, Monocytes, Fibrosis

## Abstract

**Background:**

Idiopathic pulmonary fibrosis (IPF) is a chronic fatal disease with limited therapeutic options. The infiltration of monocytes and fibroblasts into the injured lungs is implicated in IPF. Enolase-1 (ENO1) is a cytosolic glycolytic enzyme which could translocate onto the cell surface and act as a plasminogen receptor to facilitate cell migration via plasmin activation. Our proprietary ENO1 antibody, HL217, was screened for its specific binding to ENO1 and significant inhibition of cell migration and plasmin activation (patent: US9382331B2).

**Methods:**

In this study, effects of HL217 were evaluated in vivo and in vitro for treating lung fibrosis.

**Results:**

Elevated ENO1 expression was found in fibrotic lungs in human and in bleomycin-treated mice. In the mouse model, HL217 reduced bleomycin-induced lung fibrosis, inflammation, body weight loss, lung weight gain, TGF-β upregulation in bronchial alveolar lavage fluid (BALF), and collagen deposition in lung. Moreover, HL217 reduced the migration of peripheral blood mononuclear cells (PBMC) and the recruitment of myeloid cells into the lungs. In vitro, HL217 significantly reduced cell-associated plasmin activation and cytokines secretion from primary human PBMC and endothelial cells. In primary human lung fibroblasts, HL217 also reduced cell migration and collagen secretion.

**Conclusions:**

These findings suggest multi-faceted roles of cell surface ENO1 and a potential therapeutic approach for pulmonary fibrosis.

**Supplementary Information:**

The online version contains supplementary material available at 10.1186/s12931-023-02583-3.

## Background

Idiopathic pulmonary fibrosis (IPF) is a devastating and irreversible lung disease of unknown cause which has a poor median survival of only 3 ~ 5 years from the time of diagnosis [1]. Due to progressive scarring and stiffness of the lungs, IPF patients suffer from impaired gas exchange, respiratory failure, and eventually death [2]. Only two medications, nintedanib and pirfenidone, were approved so far for clinical use to slow the disease progression [2]. Discovery of novel therapeutic targets is of urgent demand for developing alternative IPF treatment.

Enolase-1 (ENO1, or alpha-enolase) is an intracellular glycolytic enzyme that catalyzes the conversion of 2-phospho-D-glycerate into phosphoenolpyruvate [3]. When translocated onto the cell surface upon inflammatory stimulation, ENO1 serves as a plasminogen receptor to localize pericellular plasminogen for its enzymatic activation [4, 5]. By binding to surface ENO1, plasminogen could be activated by urokinase-type plasminogen activator (uPA) to promote plasmin generation. Cells armed with plasmin acquire the ability of migration by proteolytically degrading the basement membrane and/or extracellular matrix [6]. Increased surface ENO1 expression on monocytes were found in patients with pneumonia, and overexpression of an ENO1 variant lacking its plasminogen binding site attenuated migration of monocytes into the inflamed lungs in mice [5]. Surface ENO1 also expressed on the surface of monocytes which mediated synovial inflammation in rheumatoid arthritis [7]. Although monocyte trafficking [8–11] and the plasminogen/plasmin axis [12, 13] were both implicated in pulmonary inflammation and fibrosis, the role of ENO1 in this context remains unknown.

Our clinical stage ENO1 blocking antibody, HL217 (previously published as HuL227), has demonstrated its anti-cancer activity via reducing cell migration in vitro and in vivo in the pre-clinical studies of prostate cancer [14]. Since fibroblast recruitment in response to lung injury also leads to fibrosis [15], we therefore hypothesized ENO1 might possess pro-fibrotic effects via facilitating monocytes and fibroblasts trafficking in lung fibrosis. Of note, in a previous report, *Sharma et al.* were the first to demonstrated that ENO1 could promote fibrosis in vitro in lung fibroblasts, in vivo in mouse model, and ex vivo in human lung tissues [16]. They discovered an anti-fibrotic strategy, using an E4 peptide (derived from the C-terminal domain of endostatin) to bind both cell surface ENO1 and urokinase plasminogen activator receptor (uPAR). Enlighted by these findings, we are investigating another anti-fibrotic strategy to intervene the ENO1/uPAR/plasmin axis by using our proprietary ENO1 blocking antibody, HL217, in this study.

Blockade of ENO1 with antibodies has been demonstrated in previous pre-clinical studies of pancreatic and lung cancer as an effective anti-invasiveness/metastasis strategy to treat cancers [17, 18]. HL217, is a humanized immunoglobulin 1 (IgG1) cross-reactive to both human and murine ENO1 (patent: US9527922B2). Herein, by blocking plasminogen receptor function of cell surface ENO1, we hypothesized HL217 may provide beneficial effects in pulmonary fibrosis via its ability to inhibit the ENO1/uPAR/plasmin axis, which is crucial for the migration of inflammatory monocytes and fibroblasts and their ensuing fibrotic activities. Our results would provide a rationale to develop ENO1 blocking antibody for treating pulmonary fibrosis.

## Methods

### Human samples

Three normal human lung formalin-fixed paraffin-embedded (FFPE) tissue sections were obtained from BioChain (#T2234152, Newark, CA, USA) and US Biomax (#HuFPT131, #HuFPT178, Derwood, MD, USA). Three human fibrotic lung FFPE sections (#CS701530, #CS702702, #CS703355) were obtained from OriGene (Rockville, MD, USA). All human blood samples from healthy donors were obtained under a protocol approved by the Institutional Review Board of the Development Center of Biotechnology (DCB) following written informed consent and ex vivo experiments were performed in accordance with the Declaration of Helsinki.

### Cell culture and reagents

Primary normal (NHLF, #CC-2512) and diseased human lung fibroblasts (DHLF-IPF, #CC-7231) were purchased from Lonza (London, UK). The cells were cultured with FGM-2 fibroblast growth medium-2 BulletKit (#CC-3132) as instructed. Cells were authenticated by Lonza, used within 15 (NHLF) or 5 (DHLF-IPF) passages, and tested negative for mycoplasma (#BSMP-101, BIOmart) throughout the study. Human Umbilical Vein Endothelial Cells (HUVEC) (#SC-8000) were from ScienCell Research Laboratories and used within 15 population doublings. Recombinant proteins of human TGF-β (#100-21) and CXCL12 (#300-28 A) were purchased from PeproTech (NJ, USA). Lipopolysaccharide (LPS) (#L2630) was purchased from Sigma (#L2630), and plasmin inhibitor tranexamic acid (TXA) (#T1810000) was from European Pharmacopoeia. HuniLife’s proprietary ENO1 monoclonal antibody (Ab) HL217 was described previously (as HuL227) for its pre-clinical investigation in prostate cancer [14]. HL217 is a humanized IgG1 and therefore human IgG1 antibodies (#HG1K, Sino Biological, PA, USA) were used as isotype control.

### Bleomycin model of pulmonary fibrosis and HL217 administration

All animals received humane care and all procedures were performed according to approved protocols from the Institutional Animal Care and Use Committee (IACUC) of TFBS Bioscience (IACUC No. TFBS2020-007 and TFBS2023-003). Eight-week-old male C57BL/6 mice were obtained from National Laboratory Animal Center (Taipei, Taiwan). The mice were anesthetized with 1.5-2% isoflurane and then a single dose of bleomycin (#B2434, Sigma, MO, USA) at 3 mg/kg (dissolved in 40 µl of PBS) was administrated intratracheally on day 0 while the mice in sham group received PBS only. HL217 at 10 mg/kg was administered intravenously with a 6-day interval from day 1. The mice in vehicle group received PBS only.

### Immunohistochemistry (IHC) staining

After deparaffinization and rehydration, lung tissue sections were performed with heat-induced epitope retrieval with 0.02 M of citrate buffer (pH 9) by microwave for 20 min. Most reagents were from Peroxidase IHC detection kit (#36,000, Peirce) unless otherwise specified. Endogenous peroxidase activity was quenched followed by blocking and then incubation with anti-ENO1 antibody (#ab227978, Abcam, 1:500) or isotype control (#ab172730, Abcam, 1:500) at 4 ^o^C for overnight. After wash, the slides were incubated with HRP-conjugated secondary antibody for 30 min and DAB reaction was performed until the desired staining was achieved subsequently. Slides were mounted after counterstained with hematoxylin. For each tissue slide, 10 random fields were acquired using a Nikon microscope (Japan) and positive stained cells were identified using software (NIS-Elements BR) with an automated threshold tool confirmed by reader’s verification.

### Histopathology

The mouse lungs were perfused with 10% formalin and stored at room temperature, processed, and embedded in paraffin. The tissue sections at 3–5 μm in thickness were stained with hematoxylin and eosin (H&E) and Masson’s trichrome stain using standard procedure. Severity of lesions was graded according to the methods described previously [19]. Degrees of lesions were graded histopathologically from one to five depending on severity (0 = not present; 1 = minimal (< 1%); 2 = slight (1–25%); 3 = moderate (26–50%); 4 = moderately severe (51–75%); 5 = severe/high (76–100%). Pulmonary fibrosis was graded as Ashcroft score according to the method described previously [20]. Each successive field was individually assessed for severity of interstitial fibrosis and allotted a score between 0 and 8 using a predetermined scale of severity. After examining the whole section, the mean score of all the fields was taken as the fibrosis score for the section and was correctively recorded to two decimal places.

### Western blotting

Lysates of human NHLF cells treated with TGF-β (1, 5, 10 ng/ml) and mouse lungs were prepared and subjected to Western blotting according to standard protocol. Primary antibodies were purchased from Abcam and listed as antigen (Cat#): ENO1 (#ab5694), fibronectin (#ab2413), α-SMA (#ab5694), and GAPDH (#ab181602).

### Collection of mice and human PBMC

EDTA-anticoagulated mice peripheral blood was collected by cardiac puncture under isoflurane anesthesia. Heparin-anticoagulated human peripheral blood was collected from healthy donors by venipuncture. After collection, the fresh whole blood was subjected to isolation of peripheral mononuclear cells (PBMC) by a density gradient centrifugation method using Ficoll Histopaque (#10831 for mice blood and #10771 for human blood, Sigma) and 15-ml SepMate tubes (#86415, Stemcell Technologies, Vancouver, Canada) according to the manufacturer’s instructions. Isolated PBMC was counted for cell numbers and used for indicated functional assays.

### Collection of BALF cells for flow cytometry

Mice were euthanized and the lungs were lavaged with PBS containing protease inhibitor cocktail (#78442, Pierce, Vienna, Austria) to collect bronchial alveolar lavage fluids (BALF). After centrifugation, the supernatant was separated for cytokine analysis, and cells were resuspended in PBS containing 2% FBS. To analyze cell surface markers, cells were first incubated with anti-CD16/32 mAb (#101302, BioLegend, San Diego, CA, USA) for 15 min at 4 ^o^C to block FcγRIIB/III, followed by incubation with the following mAbs: FITC-Ly6G (#127606, BioLegend), PE-CD11c (#117308, BioLegend), and biotin-Ly6B.2 (#MCA771GA, Bio-Rad) at 4 ^o^C for 30 min. After washing, cells were further stained with APC-streptavidin (#405207, BioLegend) at 4 ^o^C for 30 min, followed by washing and staining with 7-AAD (#420404, BioLegend). Samples were analyzed on a FACSCanto II flow cytometer (BD Biosciences, San Jose, CA, USA) using BD FACSDiva software. The percentage of cells was multiplied by the number of total cells to obtain the cell count.

### Lung tissue preparation for flow cytometry

Single cell lung tissue homogenates were performed as previously described [11, 21] with modifications. Briefly, mice were euthanized, and their lungs were perfused through the right ventricle with 10 ml PBS to remove cells in vasculature. The lungs were harvested and cut into small pieces with scissors in 1 ml digest solution (0.2 mg/ml of collagenase D (#C5138, Sigma) and 0.05 mg/ml of DNase I (#101041,59001, Roche) in RPMI-1640 (#A1049101, Gibco) supplemented with 10% FBS (#26140079, Gibco)) followed by adding 2 ml digest solution and incubated at 37 ^o^C for 1 h. The digested tissues were dissociated by using a syringe with 1.2 mm inner diameter needle and then passed through a 70 µM cell strainer. The resulting single-cell suspension was pelleted by centrifugation, resuspended with RBC lysis buffer (#555899, BD Biosciences), incubated for 5 min at room temperature. Twenty ml of PBS was added to stop the lysing procedure and cells were again pelleted by centrifugation and resuspended in staining buffer (#554656, BD). Cells were incubated with FcBlock (#553141, BD Biosciences) and stained with the following mixture of fluorochrome-conjugated antibodies (all from BD Biosciences, listed as antigen (Cat#, fluorochrome)): CD45 (#557659, APC-Cy7), Siglec-F (SigF) (#552126, PE), CD11c (#565452, BV421), CD11b (#562950, BV510), Gr-1 (#565033, PE-CY7), CD64 (#558539, Alexa 647), CD24 (#562360, PerCP-Cy5.5), and MHC-II (#562009, FITC) at 4 ^o^C for 30 min and fixed with fixation buffer (#554655, BD). Single-color controls were prepared using BD CompBeads (#552843 & #552845). Data were acquired on a BD LSRFortessa Flow Cytometer using BD FACSDiva software. Each cell population was identified using a sequential gating strategy [22]. Briefly, cells were first gated to exclude debris, then single cells were gated to select CD45^+^ cells for further analysis. Tissue-resident alveolar macrophages (TR-AM) were first gated as CD11c^+^SigF^+^ population, then neutrophils (CD11b^+^Gr-1^+^), eosinophils (CD11c^−^SigF^+^), constitutive monocytes/macrophages (Gr-1^−^MoMp) (CD11b^+^MHC-II^−^CD64^+^Gr-1^−^), classical MoMp (Gr-1^+^MoMp) (CD11b^+^MHC-II^−^CD64^+^Gr-1^+^), dendritic cells (DC) (CD11b^+^MHC-II^+^CD64^−^CD24^+^), monocyte-derived alveolar macrophage (Mo-AM) (CD11b^+^MHC-II^+^CD64^+^CD11c^+^), and interstitial macrophages (IM) (CD11b^+^MHC-II^+^CD64^+^CD11c^−^) were subsequently gated. The percentage of cells was multiplied by the number of total cells to obtain the cell count.

### Measurement of collagen and cytokine ELISA

Supernatant of BALF, cell culture, or lung tissue homogenate was collected by centrifugation and stored at -80 ^o^C until used. Collagen contents were determined by quantifying total soluble collagen using the Sircol collagen assay kit (#S5000, Biocolor, Carrickfergus, UK). Measurement of cytokines was performed using ELISA kits including mouse TGF-β (#ARG80211, Arigo Biolaboratories, Hsinchu, Taiwan), human TNF-α (#ARG80120, Arigo), human IL-1β (#ARG80101, Arigo), human IL-6 (#ARG80110, Arigo), human CCL2 (#ARG80128, Arigo), and human IL-8 (#ARG80111, Arigo). All the assays were performed according to the manufacturer’s instructions.

### Immunofluorescence (IF) staining

To stain surface ENO1, cells grown on poly-L-lysine-coated 15 mm coverslips were fixed in 4% paraformaldehyde (#47317, Alfa Aesar) for 15 min at 4 ^o^C, blocked in blocking buffer for 1 h at room temperature, and incubated with anti-ENO1 primary antibody (#ab190365, Abcam, 1:250) for overnight at 4 ^o^C. Since HL217 was developed for therapeutic purpose and not tested for application of IF staining, to ensure the reliability of the results, we used the commercially available anti-ENO1 antibody because it is suitable for IF staining as tested by the supplier. After washed with PBS, cells on the coverslips were incubated with Alexa Fluor 488 conjugated goat anti-mouse IgG secondary antibody (#A32723, Invitrogen, 1:400) for 2 h at room temperature. Unbound antibodies were washed, and cell nuclei were stained with DAPI (#D21490, Invitrogen, 2 µg/ml) for 30 min. After washed with PBS, the coverslips were mounted with Fluoromount™ Aqueous Mounting Medium (#F4680, Sigma) on glass slides. Images were acquired on a Zeiss LSM 880 confocal laser scanning microscope.

### Plasmin conversion assay

Cells were treated with LPS (1 µg/ml) for 4 h, or bleomycin (50 ng/ml) for 24 h, or TGF-β (10 ng/ml) for 4 h, washed twice with PBS, resuspended in PBS at 10^6^ cells/ml, and preincubated with 30 µM human Glu-type plasminogen (#528180, Sigma) in the absence or presence of various concentration of HL217, control hIgG1, or plasmin inhibitor TXA at at 37 °C for 1 h. After incubation, the cells were washed with PBS three times and resuspended in 100 µl PBS. Tissue plasminogen activator (tPA) at concentration of 1.5 nM (#612200, Sigma) and 0.1 mM plasmin substrate Chromogenix S-2251™ (#S820332, diapharma) were added to the cells and incubated at 37 ^o^C for 2.5 h. The plasmin activity was determined by measurement of the absorbance at 405 nm.

### Migration and chemotaxis assays

For the migration assay, after adding 900 µl of medium containing 10% FBS to the bottom well of a 24-well plate (#3464, Corning, NY, USA), cells were resuspended in medium containing 2% FBS and seeded onto the coating-free insert (#3464, Corning, 8 μm pores). For chemotaxis assay, serum-free medium was applied, and the bottom wells were supplemented with CCL2 as chemoattractant. The inserts were placed in the bottom wells and the cells were allowed to migrate at 37 °C for 18 h. The remaining cells on the upper part of the insert were removed and the cells on the lower side of the insert were fixed with methanol for 10 min, followed by staining with 1% crystal violet for additional 2 h or overnight. The insert was gently washed with PBS and dried. The bound crystal violet was eluted with 33% acetic acid and the absorbance at 590 nm was measured using a plate reader.

### Statistical analysis

Results were shown as the mean ± standard deviation, except for the mouses body weight results shown as the mean ± standard error of mean. Data analysis was performed using GraphPad Prism 8 (GraphPad Software). A two-tailed unpaired t-test was performed as appropriate to determine the significance between 2 groups. Comparison among 3 or more groups was performed using 1-way ANOVA test and corrected for multiple comparison using Tukey’s post hoc test.

## Results

### ENO1 expression is upregulated in fibrotic lungs from human and bleomycin-treated mice

**Fig. 1:**
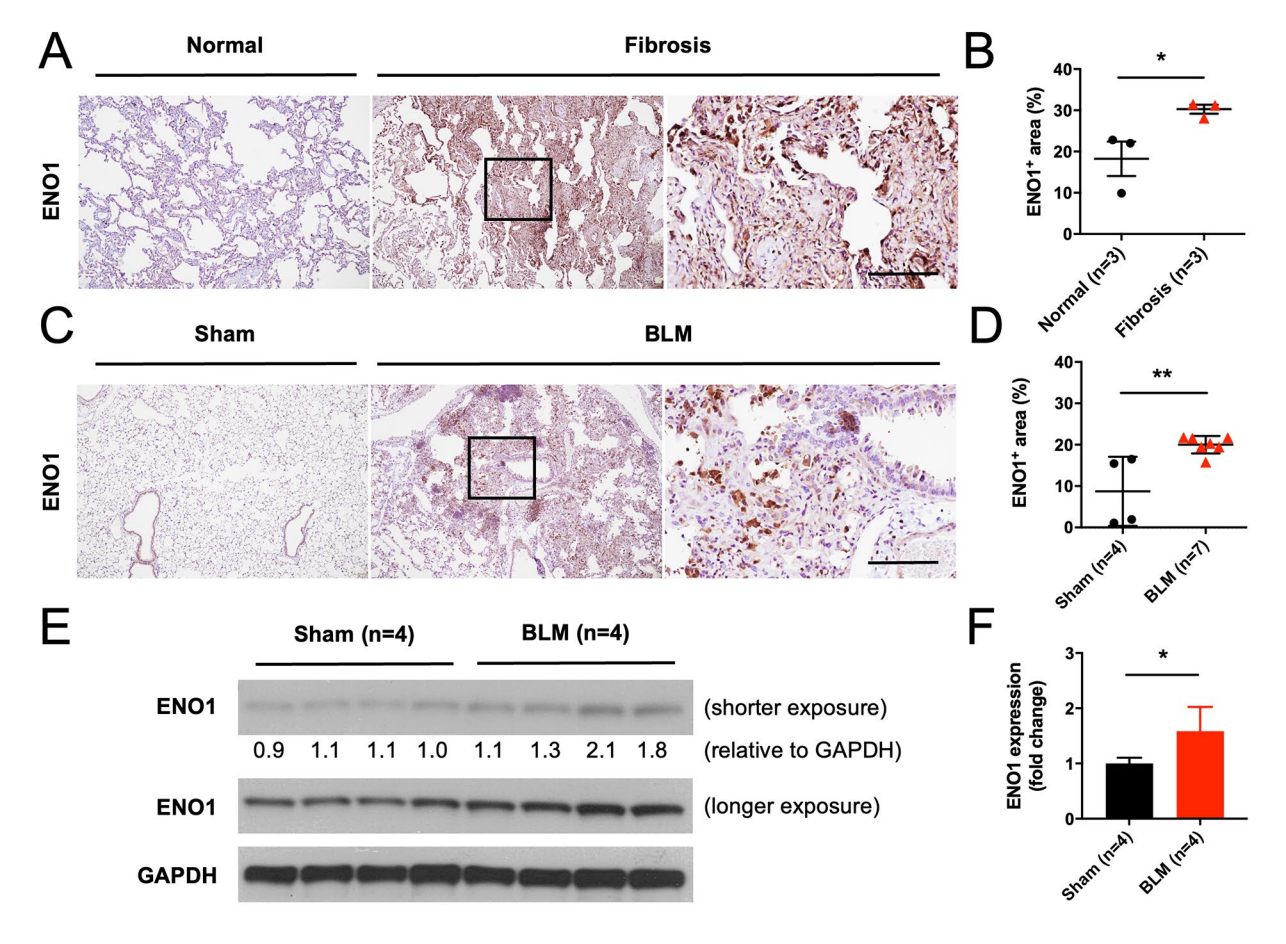
ENO1 is upregulated in fibrotic lungs from human and bleomycin-treated mice. **(A, B)** IHC staining of ENO1 was performed in commercially available human normal (n = 3) and fibrosis (n = 3) lung FFPE sections. **(C, D)** After intratracheal injection of bleomycin (BLM, 3 mg/kg) (n = 7) or PBS vehicle control (Sham) (n = 4), the mouse lungs were harvested on day 21 for preparation of FFPE sections and subjected to IHC staining of ENO1. Representative pictures **(A, C)** and quantitative results of ENO1-stained positive areas were shown **(B, D)**. After intratracheal injection of bleomycin (BLM, 3 mg/kg) (n = 4) or PBS vehicle control (Sham) (n = 4), the mouse lungs were harvested on day 21 for preparation of lysates and subjected to Western blotting for ENO1 **(E)** and the relative densitometry was shown below the representative blot after GAPDH normalization. Cropped blots were shown, and supplementary Fig. S1 presented the full-length blots. Quantitative results were shown by fold change after bleomycin treatment **(F)**. Scale bar, 100 µM. *P < 0.05, **P < 0.01. **(A, B)** One experiment was performed and each picture or data point was from one human subject. **(C-F)** Data were representative for two independent experiments. Each picture, data point, or protein band was from one mouse except (**F**) was shown as mean ± SD

To explore ENO1 expression in the context of pulmonary fibrosis, we performed ENO1 immunohistochemistry (IHC) staining in commercially available formalin-fixed paraffin-embedded (FFPE) human tissue sections of normal and fibrotic lungs. Results showed that the levels of ENO1 proteins were significantly elevated in the fibrotic lungs compared to the normal lungs (Fig. 1A, B). In bleomycin-treated mice (harvested on day 21), which is commonly used pulmonary fibrosis animal model [23], elevated expression of ENO1 in lungs was also observed compared to those from the sham group (Fig. 1C, D). Similarly, the results of Western blotting on ENO1 expression in the lung extracts also showed higher expression of ENO1 in the bleomycin-treated mice compared to the sham group (Fig. 1E, F).

### ENO1 blocking Ab HL217 ameliorates lung fibrosis of the bleomycin-treated mice

**Fig. 2:**
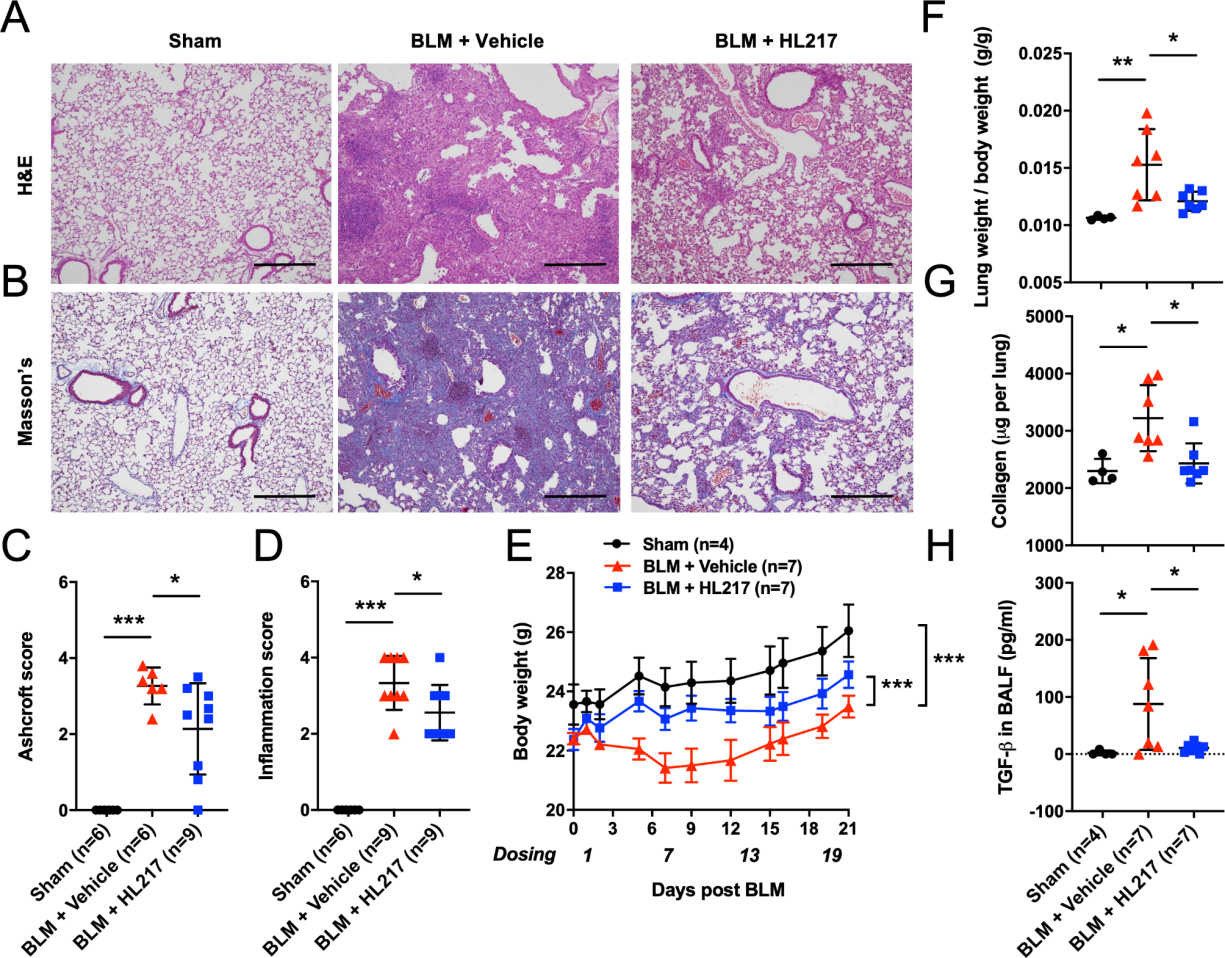
Blocking ENO1 ameliorates lung fibrosis in bleomycin-treated mice. After intratracheal injection of 3 mg/kg bleomycin (BLM) (day 0), mice were treated with ENO1 Ab HL217 (10 mg/kg) intravenously on a 6-day interval from day 1. Mouse lungs were harvested on day 14 or 21 for preparation of FFPE sections and subjected to H&E staining **(A)** and Masson’s trichrome staining **(B)**. Representative pictures on day 21 **(A, B)** and quantitative results of Ashcroft score on day 21 **(C)** and inflammation score on day 14 **(D)** were shown. Body weight change **(E)**, ratio of lung weight versus body weight **(F)**, collagen content of the lungs **(G)**, and levels of TGF-β in BALF **(H)** were shown. Scale bar, 100 µM. *P < 0.05, **P < 0.01, ***P < 0.001. Data were representative for two independent experiments. Each picture or data point was from one mouse except (E) was shown as mean ± SEM

Since ENO1 has been reported to promote fibrosis [16], we herein tested if our proprietary ENO1 Ab (HL217) could reduce fibrosis in the murine model of bleomycin-induced pulmonary fibrosis. HL217 (original name: HuL001) is an investigational antibody currently evaluated in phase I clinical trial for IPF (ClinicalTrials.gov Identifier: NCT04540770). HL217 binds specifically to human ENO1 protein with subnanomolar affinity, but not to ENO2 and ENO3. HL217 is also cross-reactive to mouse ENO1. The pharmacology activities of HL217 are to bind cell surface ENO1 and inhibit pericellular plasmin activation, subsequent plasmin-mediated migration/invasion, and pro-inflammatory cytokines production (patent: US9527922B2). The bleomycin-treated mice lungs were harvested, and the lung sections were processed with H&E (Fig. 2A) and Masson’s Trichrome staining (Fig. 2B). Results on day 21 showed a significant reduction by the treatment of HL217 (on day 1, 7, 13, and 19) on Ashcroft score which is a standardized and commonly used method of estimating severity of pulmonary fibrosis [20] (Fig. 2C). Treatment of HL217 (on day 1, 7, and 13) significantly reduced inflammation score on day 14 (Fig. 2D). The treatment of HL217 (on day 1, 7, 13, and 19) was able to significantly reduce body weight loss (absolute number in Fig. 2E and % in supplementary Fig. S2) and lung weight gain (Fig. 2F) of the bleomycin-treated mice on day 21. Deposition of collagen in lungs (Fig. 2G) and the levels of TGF-β in bronchoalveolar lavage (BALF) (Fig. 2H) on day 21 were also significantly reduced by HL217 treatment. The results suggested protective effects of ENO1 blocking Ab in pulmonary fibrosis.

### ENO1 blocking Ab HL217 reduces the recruitment of myeloid immune cells to the lungs of bleomycin-treated mice

**Fig. 3:**
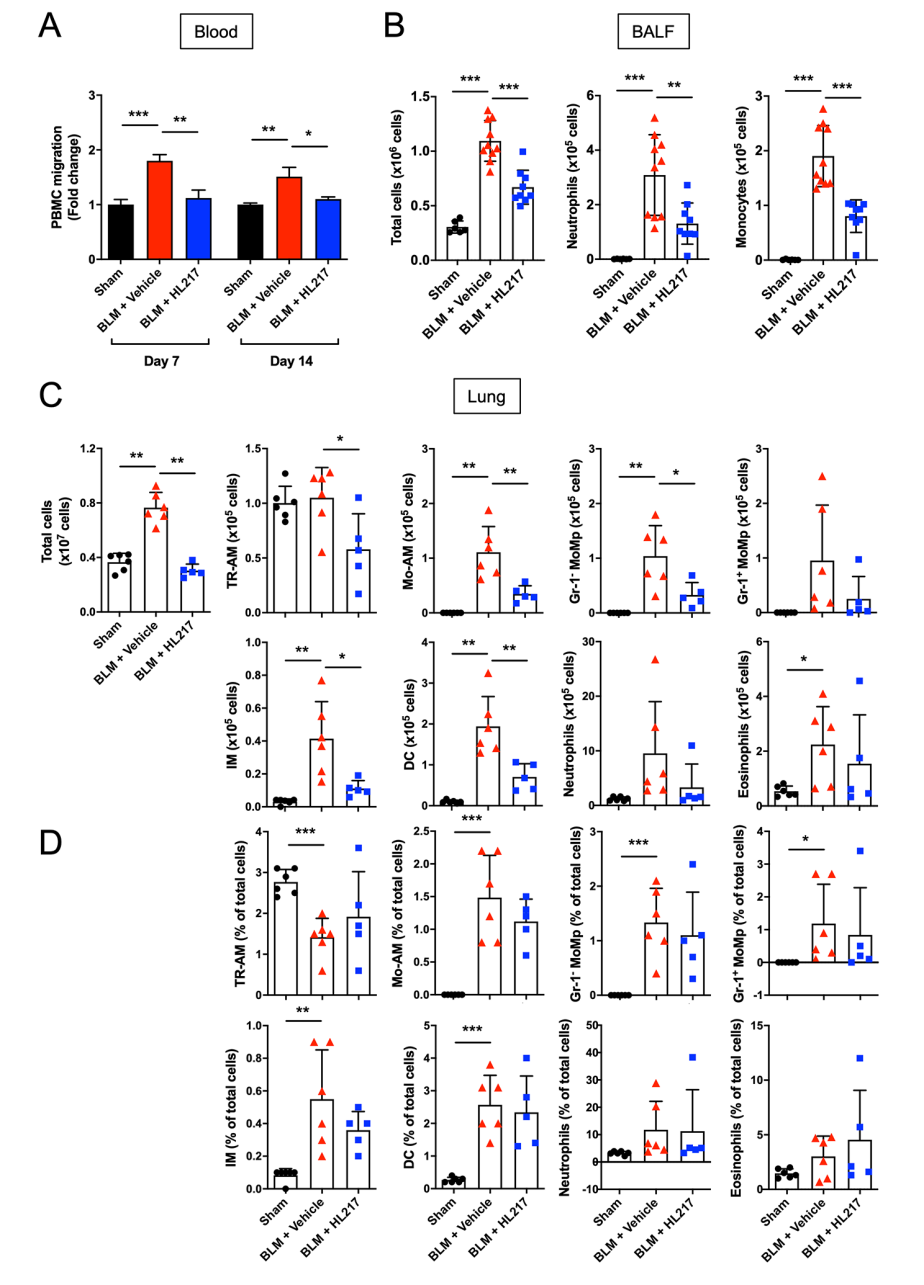
Blocking ENO1 reduced PBMC migration and immune cells recruitment to the alveolar space and lung interstitium in bleomycin-treated mice. After intratracheal injection of 3 mg/kg bleomycin (BLM) (day 0), mice were treated with ENO1 Ab HL217 (10 mg/kg) intravenously on a 6-day interval from day 1. **(A)** Pooled PBMCs of each group (n = 5) were collected on day 7 & 14 and subjected to migration assay. BALF **(B)** was collected from the groups of sham (n = 6), BLM + Vehicle (n = 10), and BLM + HL217 (n = 9) on day 4, which was then assessed by using flow cytometry for the number total cell, neutrophil (CD11c^−^/Ly6G^+^/Ly6B.2^+^ cells), or monocyte (CD11c^−^/Ly6G^−^/Ly6B.2^+^ cells). The perfused lungs **(C, D)** were collected from the groups of sham (n = 6), BLM + Vehicle (n = 6), and BLM + HL217 (n = 5) on day 7. The collected cell samples were subjected to flow cytometry analysis for tissue-resident alveolar macrophages (TR-AM) (CD45^+^CD11c^+^SigF^+^), neutrophils (CD45^+^CD11b^+^Gr-1^+^), eosinophils (CD45^+^CD11c^−^SigF^+^), constitutive monocytes/macrophages (Gr-1^−^MoMp) (CD45^+^CD11b^+^MHC-II^−^CD64^+^Gr-1^−^), classical MoMp (Gr-1^+^MoMp) (CD45^+^CD11b^+^MHC-II^−^CD64^+^Gr-1^+^), dendritic cells (DC) (CD45^+^CD11b^+^MHC-II^+^CD64^−^CD24^+^), monocyte-derived alveolar macrophage (Mo-AM) (CD45^+^CD11b^+^MHC-II^+^CD64^+^CD11c^+^), and interstitial macrophages (IM) (CD45^+^CD11b^+^MHC-II^+^CD64^+^CD11c^−^). *P < 0.05, **P < 0.01, ***P < 0.001. **(A, C, D)** Data were representative for two independent experiments. **(B)** Data were pooled from two independent experiments. Each data point was from one mouse except **(A)** was shown as fold change and each group contained three technical replicates of pooled blood from five mice

Monocytes/macrophages and neutrophils are the most studied immune cells in the context of IPF pathogenesis [24]. We therefore examined the effects of HL217 on the innate immune cells in both peripheral blood and alveolar/interstitial regions of the lungs. The migration ability of peripheral blood mononuclear cells (PBMCs) was significantly increased in bleomycin-treated mice on day 7 and 14, which was inhibited when mice received HL217 treatment (Fig. 3A). On day 4 after bleomycin challenge, there was a robust influx of inflammatory cells (primarily neutrophils and monocytes) into the alveolar space of the lungs (absolute number in Fig. 3B and % in supplementary Fig. S3), which were significantly reduced by HL217. To study the affected myeloid cells by HL217, we further investigated the various myeloid cell populations in the interstitial space of lungs from bleomycin-treated mice (absolute number in Fig. 3C and % in Fig. 3D). In summary, HL217 significantly reduced the cell number of tissue-resident alveolar macrophages (TR-AM), monocyte-derived alveolar macrophages (Mo-AM), constitutive monocytes/macrophages (Gr-1^−^MoMp), interstitial macrophages (IM), and dendritic cells (DC) in the lungs on day 7 (Fig. 3C). Except neutrophils, eosinophils, and TR-AM, the % of most infiltrated cells were upregulated in response to bleomycin challenge, which however were not significantly affected by HL217 treatment (Fig. 3D). Significant reduction of HL217 on the cell number of TR-AM might not be relevant to alleviation of lung fibrosis, since previous report showed depletion of TR-AMs had no effect on lung fibrosis [10]. Taken together, HL217 reduced the numbers of infiltrating cells to the lung interstitium but without altering their ratio of composition. The results together suggested ENO1 regulates the trafficking of multiple innate immune cells to the lungs in response to bleomycin challenge.

### ENO1 blocking Ab HL217 reduces lipopolysaccharide (LPS)-induced plasmin activation, migration, and cytokines secretion of human PBMC

**Fig. 4:**
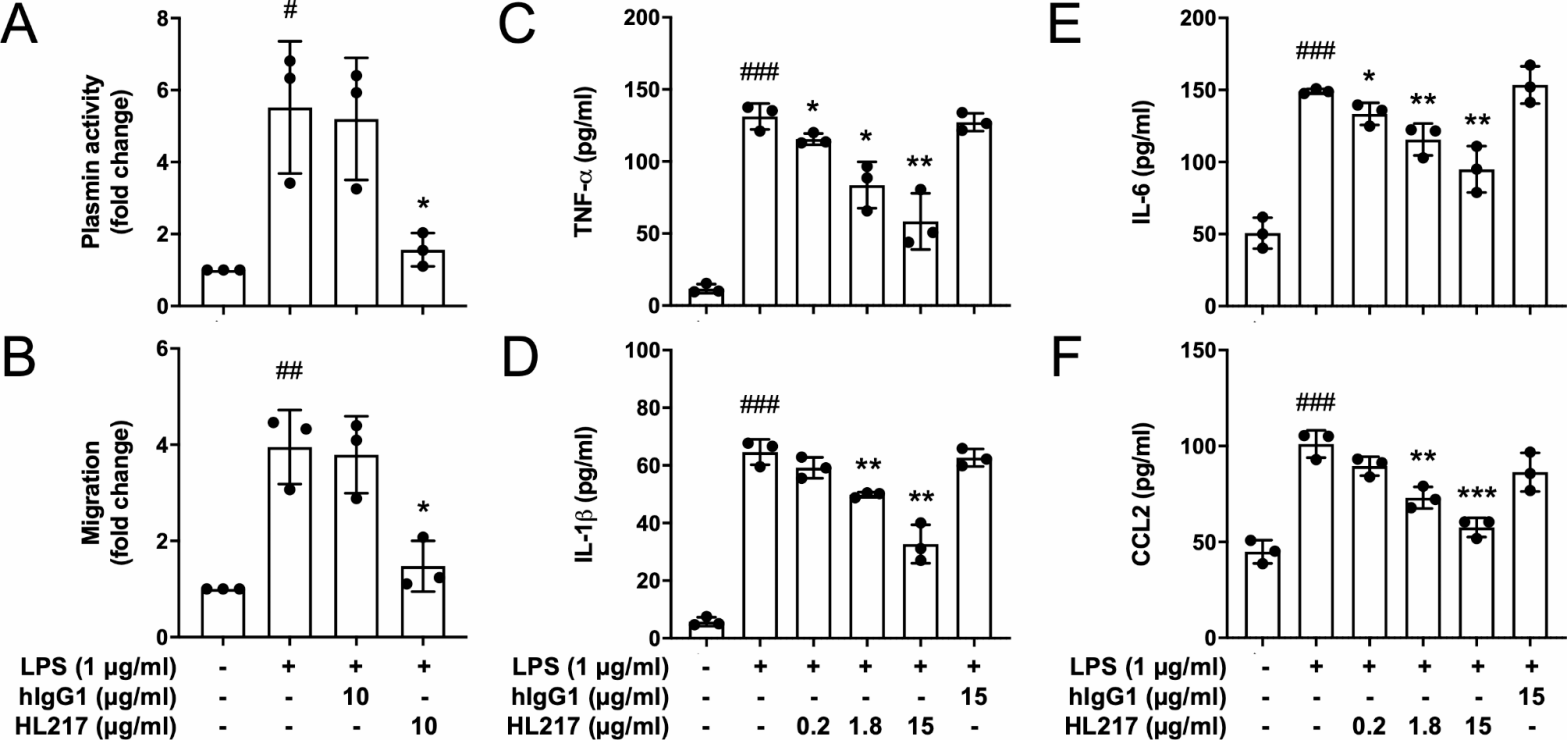
Anti-inflammatory effects of ENO1 Ab HL217 in LPS-stimulated primary human PBMC. Fresh peripheral blood was collected from 3 healthy donors and added with indicated concentrations of LPS, control human IgG1 (hIgG1), and HL217 for 4 h, followed by isolation of PBMC. The isolated PBMC was subjected to measurement of cell-associated plasmin activity **(A)** and cell migration **(B)**. **(C-F)** PBMC was isolated from 3 healthy donors and treated with indicated concentrations of LPS, hIgG1, and HL217 for 24 h. Cell culture supernatants were collected and subjected to measurement of pro-inflammatory cytokines, including TNF-α **(C)**, IL-1β **(D)**, IL-6 **(E)**, and CCL2 **(F)**, by ELISA. ^#^*P* < 0.05, ^##^*P* < 0.01, ^###^*P* < 0.001 vs. untreated cells; *P < 0.05, **P < 0.01, ***P < 0.001 vs. the LPS-treated group. Pooled data of three independent experiments and each data point was from one human subject

Previous study showed LPS induces ENO1 translocation to cell surface of PBMC and monocytes, which further mediates plasmin activation and migration [5, 25]. Moreover, plasmin has been shown as a potent proinflammatory cell activator [26] to induce cytokine secretion via triggering AP-1 and NF-κb-mediated signaling [27] or p38 MAPK and janus kinase (JAK)/STAT pathways [28]. To relate the anti-inflammatory effects of HL217 in mice to human, we then studied plasmin activity, cell migration, and cytokine production upon HL217 treatment, using LPS-stimulated human blood as ex vivo model [5]. Human blood from 3 healthy donors was stimulated with LPS in the presence or absence of HL217 and control antibody (hIgG1) for 4 h followed by isolation of PBMC. Plasmin activity (Fig. 4A) and cell migration (Fig. 4B) were measured in the isolated PBMC. Results showed HL217 significantly reduced LPS-induced plasmin activation and cell migration. In another experiment, isolated PBMC from 3 healthy donors was simulated with LPS in the presence or absence of control antibody or increasing concentrations of HL217 for 24 h. Culture supernatants were collected and measured for inflammatory cytokines. We found HL217 could dose-dependently reduce secretion of TNF-α, IL-1β, IL-6, and CCL2 in the LPS-stimulated human PBMC (Fig. 4C-F). The anti-inflammatory effects of HL217 found in human PBMC were in line with the findings (Fig. 3) in bleomycin-treated mice.

### ENO1 blocking Ab HL217 reduces bleomycin-induced plasmin activation and chemokines secretion of primary human endothelial cells

**Fig. 5:**
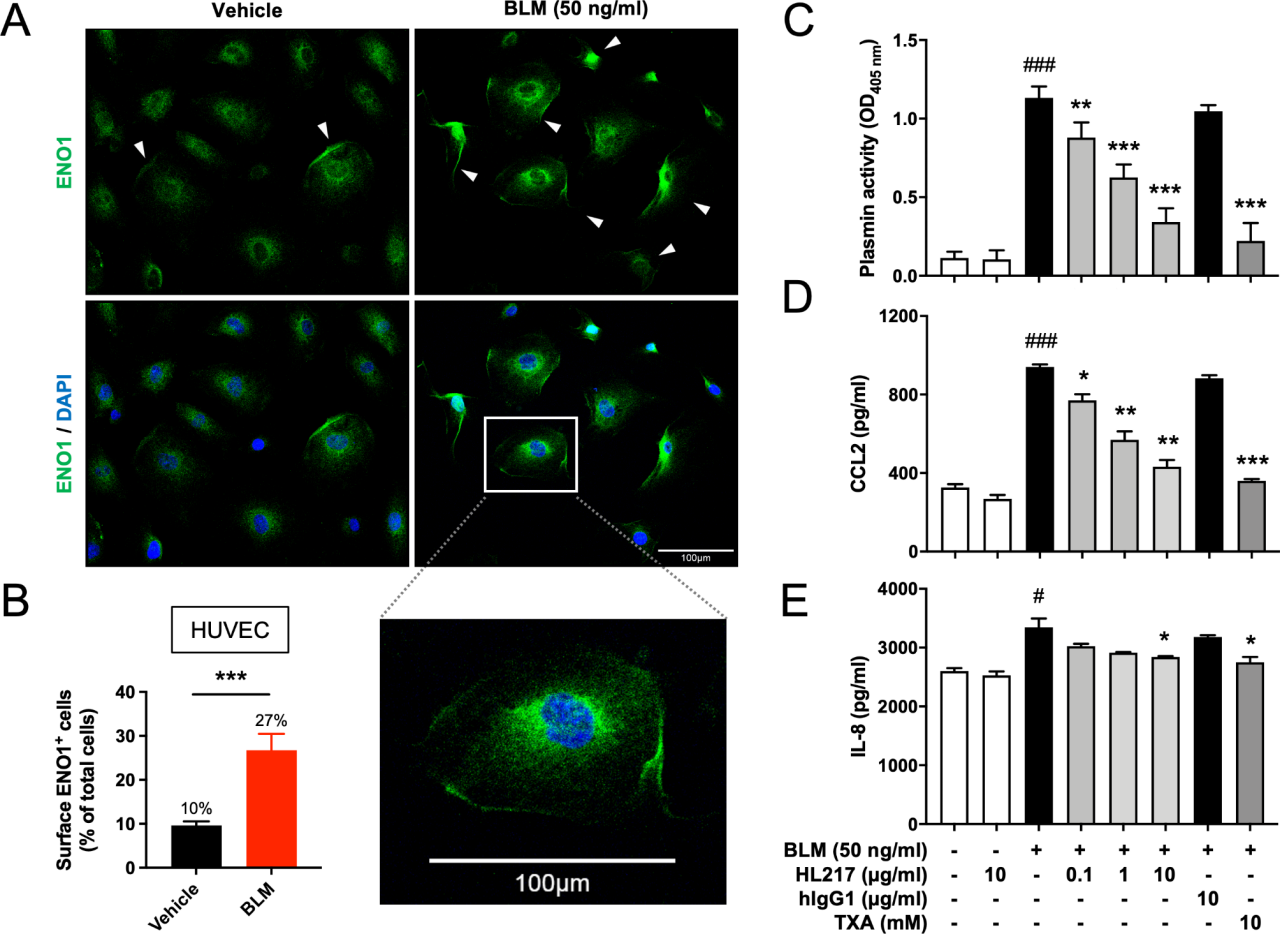
Anti-inflammatory effects of ENO1 Ab HL217 in bleomycin-stimulated primary human endothelial cells (HUVEC). Primary human umbilical vascular endothelial cells (HUVEC) were treated with 50 ng/ml bleomycin (BLM) for 24 h and subjected to the measurement of surface ENO1 expression **(A, B)** by using immunofluorescence staining. **(A)** Representative pictures of 4 independent experiments were shown. Cells expressing surface ENO1 were indicated with white arrowhead in the upper panel (ENO1 expression shown in green). The lower panel (merged with nuclear staining DAPI to indicate location of all cells) was kept without arrowhead for clarity. Cells expressing surface ENO1 were manually counted in 5 area of one high power field picture. Four pictures of each group were taken and counted. **(B)** Percentages (%) of surface ENO1 expressing cells was determined over the number of all nucleated cells. **(C-E)** HUVEC were treated with indicated concentrations of BLM, HL217, hIgG1, and plasmin inhibitor tranexamic acid (TXA) for 24 h. Cell-associated plasmin activity was measured **(C)** and cell culture supernatants were collected for measurement of chemokines, including CCL2 **(D)** and IL-8 **(E)**, by ELISA. Scale bar, 100 µM. ^#^*P* < 0.05, ^###^*P* < 0.001 vs. untreated cells; *P < 0.05, **P < 0.01, ***P < 0.001 vs. the BLM-treated group. Pooled data of three independent experiments were shown as mean ± SD (one technical replicate and three biological repeats per group)

To address the possible mechanism by which HL217 reduced the recruitment of innate immune cells to the injured lungs, we used primary human umbilical vascular endothelial cells (HUVEC) as an in vitro model, since endothelial cell injury is believed to be an initiating event preceding the development of fibrosis [29]. Endothelial cells secrete chemo-attractants in response to fibrotic stimuli [30] and plasmin [26], which may contribute to the recruitment of immune cells into the lungs. We treated HUVEC with bleomycin and found significant increase of surface ENO1 expression (Fig. 5A, B) but not total ENO1 expression (supplementary Fig. S4), and plasmin activation which could be dose-dependently reduced by HL217 and plasmin inhibitor tranexamic acid (TXA) (Fig. 5C). TXA is a lysine analogue which binds to plasminogen and prevents plasmin activation [31]. Bleomycin treatment also induced the secretion of monocyte-recruiting chemokine CCL2 (Fig. 5D) and neutrophil-recruiting chemokine IL-8 (Fig. 5E), which were both reduced by HL217 in a dose-dependent manner and by TXA. In contrast, human IgG1 had no effect on all plasmin-induced activities. Results suggested an anti-inflammatory mechanism of HL217 on injured endothelial cells via inhibition of ENO1-mediated plasmin activation and ensuing cell recruitment.

### Surface ENO1 mediates the migration and chemotaxis of primary human lung fibroblasts

**Fig. 6:**
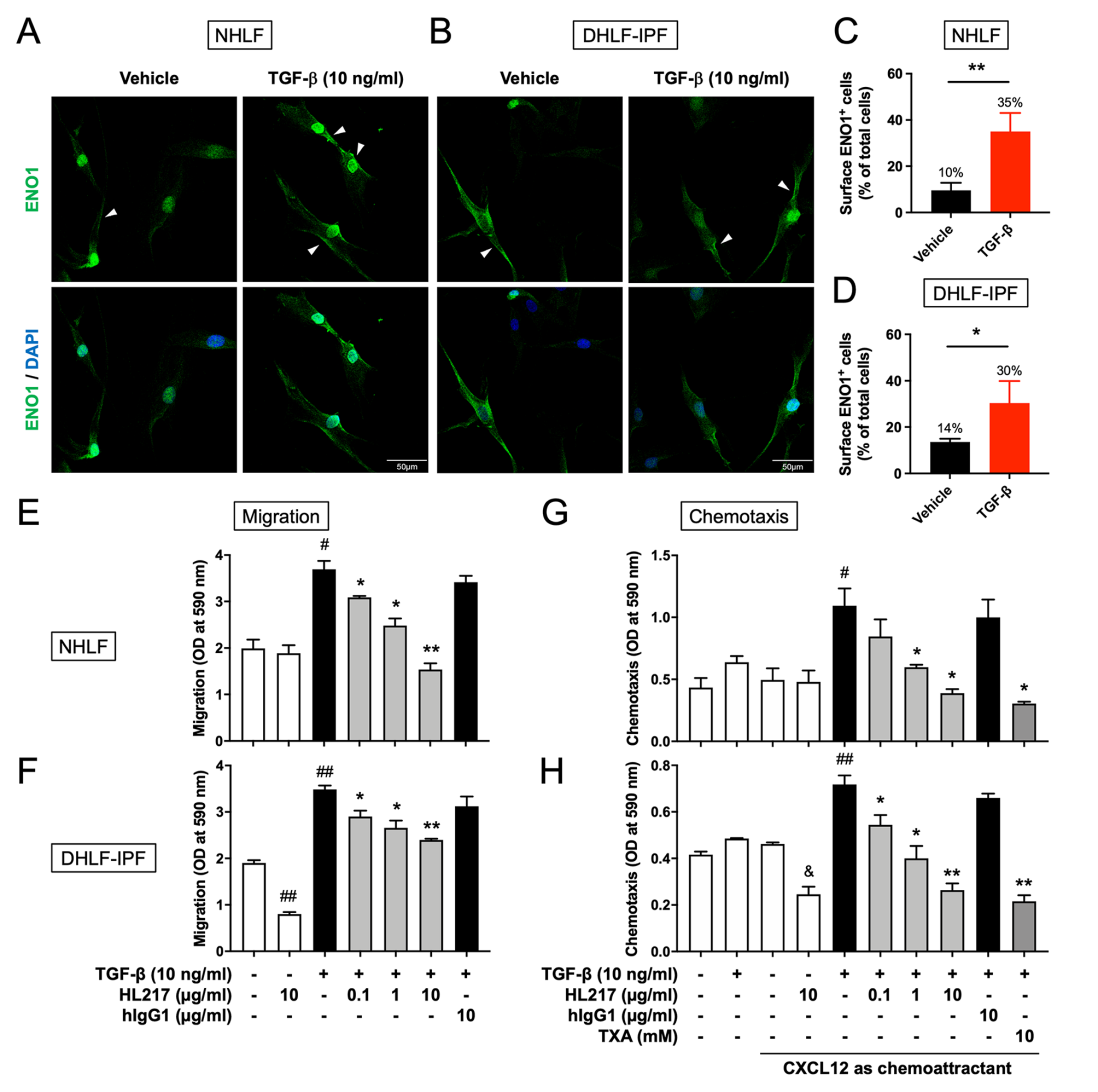
Anti-fibrotic effects of ENO1 Ab HL217 in TGF-β-stimulated primary human lung fibroblasts. Primary normal human lung fibroblasts (NHLF) **(A, C)** or diseased human lung fibroblasts from IPF patient (DHLF-IPF) **(B, D)** were treated with 10 ng/ml TGF-β for 24 h and subjected to the measurement of surface ENO1 expression by using immunofluorescence staining. **(A, B)** Representative pictures of 4 independent experiments were shown. Cells expressing surface ENO1 were indicated with white arrowhead in the upper panel (ENO1 expression shown in green) and lower panel (merged with nuclear staining DAPI to indicate location of all cells) was kept without arrowhead for clarity. Cells expressing surface ENO1 were manually counted in 5 area of one high power field picture. Four pictures of each group were taken and counted. **(C, D)** Percentages (%) of surface ENO1 expressing cells was determined over the number of all nucleated cells. NHLF **(E, G)** and DHLF-IPF **(F, H)** were treated with 10 ng/ml TGF-β for 4 h and allowed to migrate for another 18 h in the absence or presence of indicated concentrations of HL217, hIgG1, or TXA in the migration assay **(E, F)** or the CXCL12 chemotaxis assay **(G, H)**. ^#^*P* < 0.05, ^##^*P* < 0.01 vs. untreated group; *P < 0.05, **P < 0.01 vs. TGF-β-treated group (**E**, **F**) or TGF-β- and CXCL12-treated group (G, H); ^&^*P* < 0.05 vs. the group with only CXCL12 as chemoattractant. Pooled data of two independent experiments were shown as mean ± SD (one technical replicate and one biological repeat per group for each experiment)

Besides the infiltration of innate immune cells, interstitial fibroblasts also migrate to the site of injured tissues for wound healing and tissue repair [32]. However, the repairing system is excessive in IPF patients causing abnormal scarring of the lungs. To test our hypothesis again that surface ENO1-mediated cell migration of the involved lung fibroblasts is intrinsic and crucial in the development of pulmonary fibrosis, we investigated the in vitro effect of HL217 on the migration ability of primary human lung fibroblasts. We used commercially available primary human lung fibroblasts isolated from normal control (NHLF) and IPF patient (DHLF-IPF) as in vitro models owing to their roles as responders and producers of fibrotic mediators [33]. Similar with HUVEC, no significant increase of total ENO1 expression in NHLF in response to bleomycin treatment (supplementary Fig. S5). But as expected, TGF-β stimulated significant surface ENO1 expression in both NHLF and DHLF-IPF (Fig. 6A-D). The migration of either NHLF or DHLF-IPF was dose-dependently reduced by HL217 but not by hIgG1 (Fig. 6E, F). Since fibroblasts were known to be attracted by chemokine CXCL12 and may contribute to the development of lung fibrosis [34–36], we performed the chemotaxis assay and found consistent results that HL217 dose-dependently reduced TGF-β-stimulated migration responding to CXCL12 in both NHLF and DHLF-IPF (Fig. 6G, H). It was interesting to observe that HL217 was able to inhibit cell migration of DHLF-IPF even in the absence of TGF-β without CXCL12 (Fig. 6F) or with CXCL12 (Fig. 6H) as chemoattractant, possibly due to higher surface ENO1 expression if compared to NHLF (Fig. 6A-D). Plasmin inhibitor TXA, but not hIgG1, also showed similar inhibitory effects as HL217, which together suggested an anti-fibrotic mechanism of HL217 on inhibition of ENO1-mediated plasmin activation and cell migration of lung fibroblasts in response to fibrotic stimulation.

### Blocking ENO1 reduces collagen secretion of primary human lung fibroblasts

**Fig. 7:**
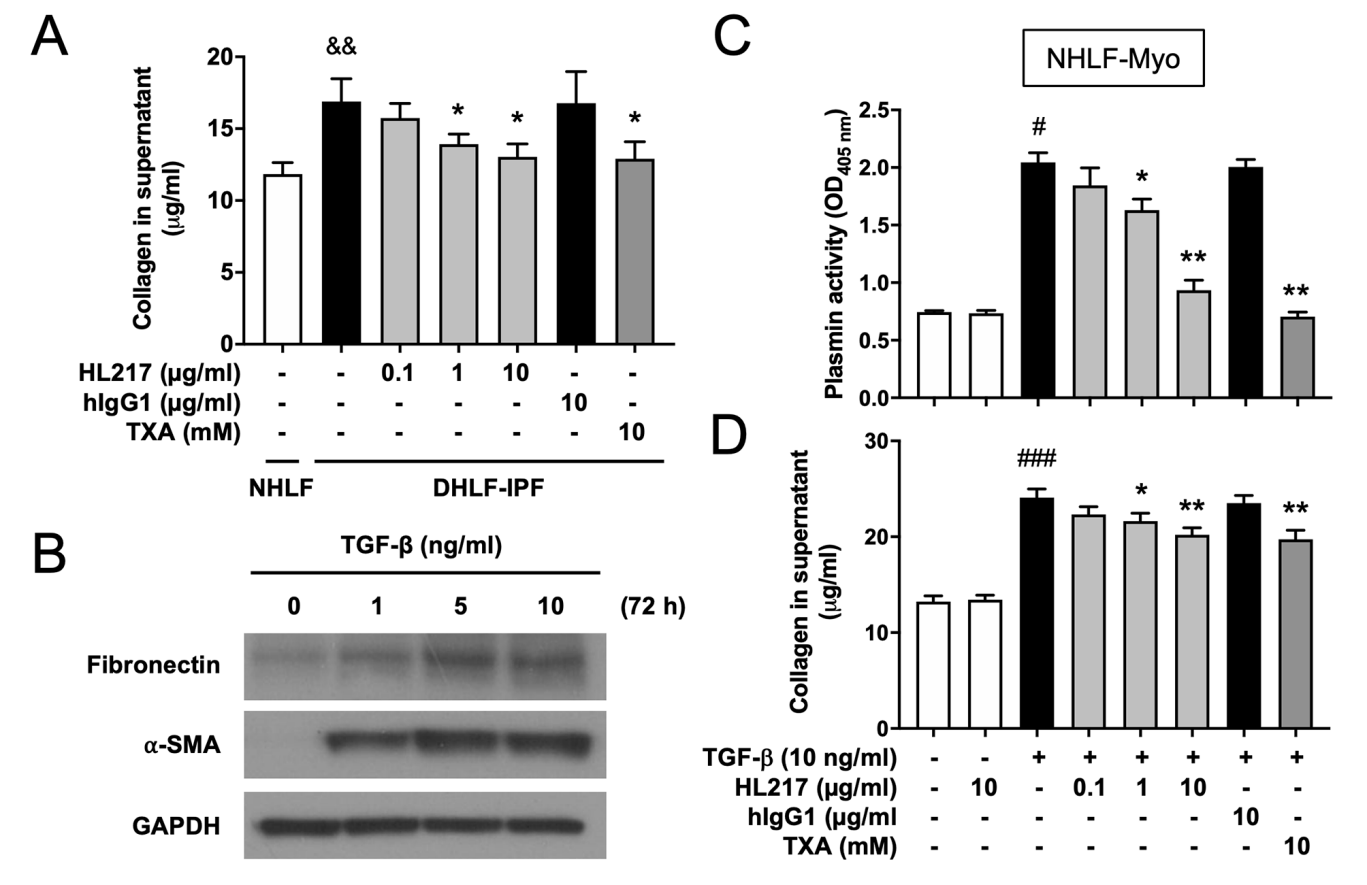
Blocking ENO1 reduces plasmin activation and collagen secretion in primary human lung fibroblasts. **(A)** DHLF-IPF was allowed to grow for 48 h in the absence or presence of indicated concentrations of HL217, hIgG1, or TXA. Untreated NHLF was cultured as control. The cell culture supernatants were collected and subjected to the measurement of collagen by using Sircol assay. **(B)** Primary normal human lung myofibroblasts (NHLF-Myo) were differentiated from NHLF by treatment with TGF-β for 72 h. Increasing expression of fibronectin and α-SMA confirmed the differentiation of NHLF-Myo. Cropped blots were shown, and supplementary Fig. S7 presented the full-length blots. **(C)** The NHLF-Myo was treated with TGF-β for another 4 h and subjected to the measurement of cell-associated plasmin activity in the absence or presence of indicated concentrations of HL217, hIgG1, or TXA. **(D)** The cell culture supernatants were collected and subjected to the measurement of collagen by using Sircol assay. ^#^*P* < 0.05, ^###^*P* < 0.001 vs. the untreated group; ^&&^*P* < 0.01 vs. the NHLF group; *P < 0.05, **P < 0.01 vs. the TGF-β-treated group. Pooled data of three independent experiments were shown as mean ± SD (one technical replicate and three biological repeats per group) except (**B**) was representative for two independent experiments (each protein band was from one biological repeat)

To elucidate whether HL217 possesses direct anti-fibrotic effects, we examined the effects of HL217 on spontaneous collagen secretion of DHLF-IPF or TGF-β-induced collagen secretion of myofibroblasts. We found the collagen secretion was inherently higher in DHLF-IPF than in NHLF when cultured for 48 h in the absence of TGF-β, which could be reduced by HL217 as well as TXA (Fig. 7A). Then, lung myofibroblasts (NHLF-Myo) were differentiated by treating NHLF with TGF-β for 72 h and confirmed for the elevated expression of fibronectin and α-smooth muscle actin (α-SMA) (Fig. 7B). We treated NHLF-Myo with TGF-β for another 4 h to detect plasmin activation (Fig. 7C) or for another 48 h to detect collagen secretion (Fig. 7D). We found HL217 dose-dependently reduced plasmin activation and collagen secretion, similar with plasmin inhibitor TXA but not hIgG1. The results were in line with the previous report by *Sharma et al.*, demonstrating that treatment with recombinant ENO1 protein induced collagen and fibronectin secretion in lung fibroblasts [16]. Taken together, our results indicated pro-inflammatory and pro-fibrotic roles of cell surface (or extracellular) ENO1 in pulmonary fibrosis, which might provide rationales to explain the efficacy of HL217 observed in bleomycin-treated fibrotic mice.

## Discussion

To our knowledge, this is the first study demonstrating that ENO1 targeting antibody could be used as a therapeutic strategy for pulmonary fibrosis. In brief, targeting ENO1 by our proprietary antibody HL217 ameliorates bleomycin-induced pulmonary fibrosis in vivo as evidenced by the attenuation of fibrosis (i.e., collagen deposition in lungs), TGF-β reduction in BALF, and immune cells infiltration in lungs. Moreover, the results of in vitro studies provide scientific evidence and rationales for the test of HL217 in human disease. The pathogenesis of lung fibrosis is undoubted complex and interplayed with multifaceted factors, including various cell types, cytokines, chemokines, and fibrotic mediators. In this study, we demonstrated that ENO1 is upregulated in both human and mouse fibrotic lungs. Herein, we propose that surface ENO1 could play an important role in lung fibrosis based on the following evidence: HL217 has an inhibitory effect on the ENO1/uPAR/plasmin axis, which in turn governs cell trafficking (human PBMC, endothelial cells, lung fibroblasts and myofibroblasts, monocytes, neutrophils), inflammatory mediators (TNF-a, IL-6, IL-1b, CCL2, IL-8), and fibrotic mediators (TGF-β, collagen). Most interestingly, the presence of surface ENO1 and plasmin activation, if not only, seem to drive the above different cell populations toward the fibrotic process.

*Sharma et al.* were the first to show that ENO1 can promote lung fibrosis [16]. They found silencing ENO1 expression downregulated fibrosis-related proteins, such as COL1α1 (collagen type I alpha 1 chain) and fibronectin, in TGF-β1-stimulated normal lung fibroblasts or diseased lung fibroblasts derived from systemic sclerosis patients. Whereas overexpression of ENO1 or transfection with recombinant ENO1 protein (rENO1) could induce pro-fibrotic phenotype of primary lung fibroblasts. Notably, intratracheal administration of rENO1 induced lung fibrosis in mice and treating human lung tissue cores with rENO1 also significantly increased expression of various fibrosis proteins. Although we have showed that ENO1 was upregulated in human and mouse fibrotic lungs, we do not actually know if “surface” ENO1 (vs. cytosolic) is upregulated or not, due to the technical limitation of immunohistochemistry staining. Besides, the sample size of this result was relatively small. Whether and where the surface ENO1 is overexpressed in IPF patients await further verification. But the results of *Sharma et al.* shed light on the roles of extracellular ENO1 in lung fibrosis. Taken together our study using ENO1 Ab, extracellular (or surface) ENO1 might be potential therapeutic target. We could reason and speculate that the target cells of ENO1 Ab HL217 are endothelial cells, neutrophils, circulating monocytes, alveolar macrophages, and lung fibroblasts/myofibroblasts in the context of pulmonary fibrosis. One evidence is that the pharmacology effects of HL217, surface ENO1 specific, is observed and apparent for the abovementioned cell populations. Co-localization of ENO1 expression with CD45^+^ leukocytes or α-SMA^+^ myofibroblasts could be found in the lungs of bleomycin-treated mice (supplementary Fig. S6). Little co-localization of ENO1 to CD31 expression (supplementary Fig. S6B) disagreed with in vitro results shown in Fig. 5. We speculated endothelial cells injury might occur in earlier phase even ahead of inflammation, and time dependent ENO1 expression on endothelial cells needs to be investigated. Although our results clearly indicate the involvement of surface ENO1 in bleomycin-induced lung fibrosis, we have not optimized the dosing schedule of HL217 as a “treatment” protocol. Our preliminary results showed HL217 (administrated on day 9, 14, and 19, for a treatment purpose) significantly suppressed lung collagen deposition (data not shown). Further studies with various therapeutic regimens are needed to justify the use of HL217 in clinical trials. Nevertheless, this study is the first to suggest ENO1 Ab with therapeutic potential for pulmonary fibrosis.

The roles of monocytes in IPF have recently gained increasing attention due to the observation that elevated monocyte count was associated with increased risks of IPF progression and mortality [37, 38]. Previous studies also demonstrated depletion of Ly6C^hi^ circulating monocytes or alveolar macrophages [8], or knockout of the *Ccr2* gene [9], all reduced pulmonary fibrosis in animal models. More specifically, depletion of monocyte-derived alveolar macrophages (Mo-AMs) within an injured lung can lessen the severity of fibrosis [10]. Knockout of ENO1 interacting protein B7H3 [39] has been shown to be protected from bleomycin-induced lung fibrosis via reduced expansion of activated Ly6C^hi^ monocytes in bone marrow [40]. It is known that ENO1 translocates to the surface of monocytes and promote cell migration during inflammation [5], and we herein provide a feasible strategy to attenuate monocytes and Mo-AMs recruitment to the injured lungs and thus to protect the lungs from further fibrotic process. Furthermore, fibroblasts/myofibroblasts are long recognized as key players in fibrosis not only in lung but also liver, kidney, and even in cancer [41]. ENO1 as an autoantigen has been reported in systemic sclerosis (SSc)-associated interstitial lung disease [42] and liver fibrosis [43] evidenced as the frequent presence of anti-ENO1 autoantibodies in these patients. The role of ENO1 in promoting fibrosis was first demonstrated by *Sharma et al.* in recombinant ENO1-treated human lung fibroblasts and lung tissue cores and also in ENO1-silenced SSc lung fibroblasts [16]. Since surface ENO1 is widely expressed on key cell populations involved in fibrosis, we believe investigation of HL217 in other fibrotic diseases would be of great value.

There were ample evidence addressing the involvement of plasminogen activation in pulmonary fibrosis [12]. Targeting matrix metalloproteinases (MMPs) [44] or protease activated receptors (PAR-1/PAR-2) [45, 46] have been implicated in IPF treatment. Since plasmin is upstream of activation of MMPs [47], PARs [48], and cell-associated latent TGF-β [49], we therefore hypothesize that the main action mechanism of HL217, if not only, is cell-associated plasmin activity-dependent. Tranexamic acid (TXA) is a plasmin inhibitor and previously used in clinical practice as an antifibrinolytic agent (40), which binds to circulating plasminogen and prevents plasminogen from binding to fibrin. Prevention of binding to fibrin inhibits the conversion of plasminogen to plasmin by fibrin-bound tissue type plasminogen activator (tPA). Although both TXA and HL217 have similar antifibrinolytic activity, their mechanisms and interacting partners are not the same. HL217, however, only binds to cell surface ENO1, not to plasminogen, and thus inhibits only cell-associated plasmin activity without interfering with the fibrinolysis system, i.e. not interfering with the binding of plasminogen to fibrin. Given the different hypothesized roles of plasmin, spatial regulation of plasmin in lung fibrosis has been discussed [48]. In the airspace of the lung, plasmin activity is low, and fibrin accumulated. On the other hand, in the lung interstitium, plasmin activity is high and contribute to fibrotic lesions of damaged parenchymal tissues. Therefore, global inhibition of plasmin might not be all beneficial for pulmonary fibrosis. Our results may provide a strategy to target only surface ENO1 on the cells in the lung interstium, including endothelial cells, infiltrated immune cells, and fibroblasts. However, the roles of intracellular ENO1 might be quite different from surface ENO1, which is involved in the trans-differentiation of alveolar epithelial type II (ATII) cells to type I (ATI) cells upon injury (41).

The functions of ENO1 are highly determined by spatial regulation [50]. The cytosolic catalytic function of ENO1 participates in glycolysis (or Warburg effect in cancer) and the extracellular function of ENO1 involved in plasmin-mediated pericellular proteolysis might both contribute to the lung fibrotic process. Accumulating reports implicated glycolytic reprogramming in lung fibrosis [51–55]. *Sharma et al.* clearly demonstrated the silencing of ENO1 expression in primary fibroblasts downregulated profibrotic genes expression, and ENO1 protein (extracellular) promoted a fibrotic phenotype in vivo and ex vivo [16], which suggest both cytosolic and extracellular roles for ENO1 in promoting fibrosis. In agreement, our results further support the pathologic roles of extracellular/cell surface ENO1 in mediating lung fibrosis.

IPF is a complex disease, and we believe that a good therapeutic strategy should be multifactorial intervention rather than a single mechanism approach. This study demonstrates that surface ENO1 regulates cell trafficking, inflammatory mediators, and fibrotic mediators involving various pathological cell populations in pulmonary fibrosis. These experimental results also suggest the first-in-class ENO1 monoclonal antibody has potential to be developed as an anti-fibrotic therapeutic agent.

### Electronic supplementary material

Below is the link to the electronic supplementary material.

Supplementary Material 1

## Data Availability

All data generated or analyzed during this study are included in this published article.
