# Supplementary material for: Monoclonal enolase-1 blocking antibody ameliorates pulmonary inflammation and fibrosis
Source: Respir Res. 2023 Nov 14;24:280. doi: 10.1186/s12931-023-02583-3 (PMC10647181; doi:10.1186/s12931-023-02583-3)
Supplement: Supplementary file 1 — Supplementary Material 1 [file 12931_2023_2583_MOESM1_ESM.pdf]

Supplementary Materials

A

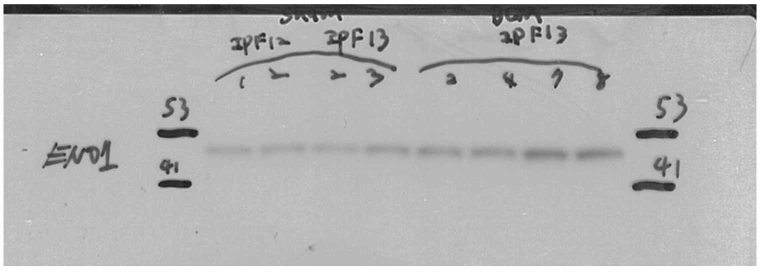

B

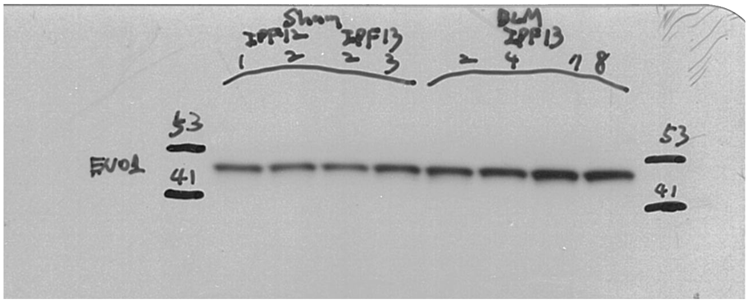

C

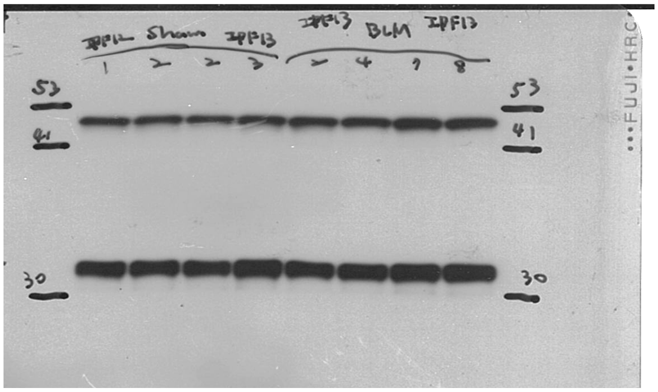

Fig. S1. Full length blots of Figure 1E

Proteins were separated by 10% SDS-PAGE. (A) for ENO1 with shorter exposure, (B) for ENO1 with longer exposure, and (C) for GAPDH (lower band). ENO1 and GAPDH are the same membrane that has undergone two different primary antibody incubations respectively. The above band in (A) and (C) are used for statistics in Figure 1F.

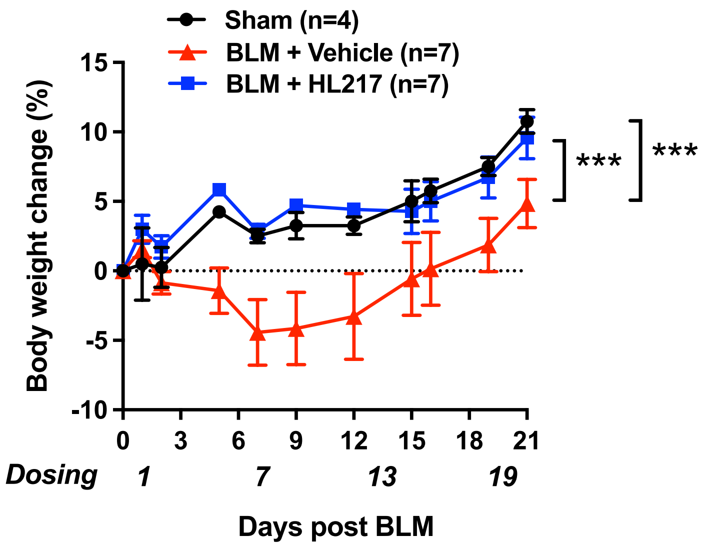

Fig. S2. HL217 attenuates bleomycin-induced body weight loss.

After intratracheal injection of 3 mg/kg bleomycin (BLM) (day 0), mice were treated with ENO1 Ab HL217 (10 mg/kg) intravenously on a 6-day interval from day 1. Body weight was monitored, and the percentage of change was shown as compared to individual body weight on day 0. ****P* <0.001 was calculated using repeated-measures one-way ANOVA.

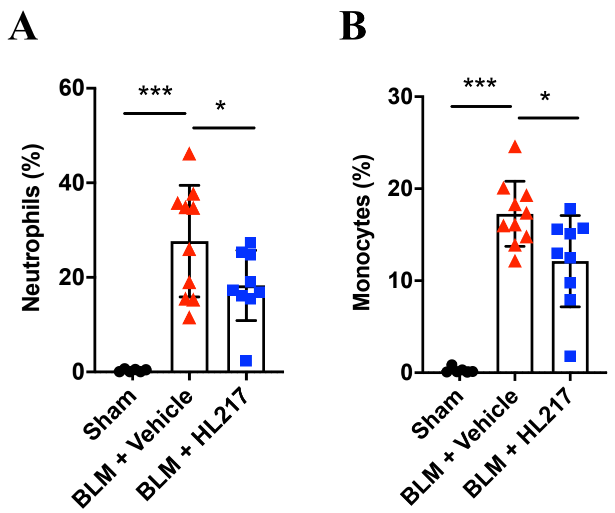

Fig. S3. Percentages of neutrophils and monocytes in BALF.

After intratracheal injection of 3 mg/kg bleomycin (BLM) (day 0), mice were treated with a single dose of ENO1 mAb HL217 (10 mg/kg) intravenously on day 1. BALF was collected on day 4 from the groups of Sham (n=6), BLM+Vehicle (n=10), and BLM+HL217 (n=9). The cells in BALF were subjected to flow cytometry analysis for indicated cell populations. **P* <0.05, ****P* <0.001

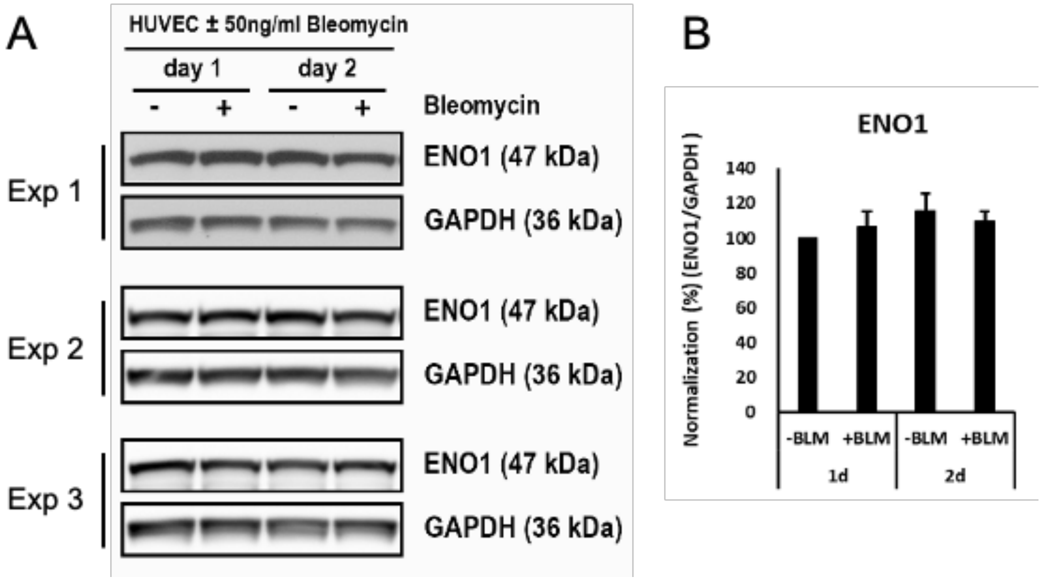

Fig. S4. No significant change of total ENO1 protein expression of HUVEC in response to bleomycin treatment.

HUVEC cells were treated with 50 ng/ml of bleomycin for 1 or 2 days and protein lysates were separated by 10% SDS-PAGE to determine the expression levels of ENO1. (A) Blots from 3 independent experiments (Exp 1~3) were shown. GAPDH was used as loading control. ENO1 and GAPDH are the same membrane that has undergone two different primary antibody incubations respectively. (B) Densitometry was performed, and quantitative results were shown by % to untreated cells on day 1 (100%).

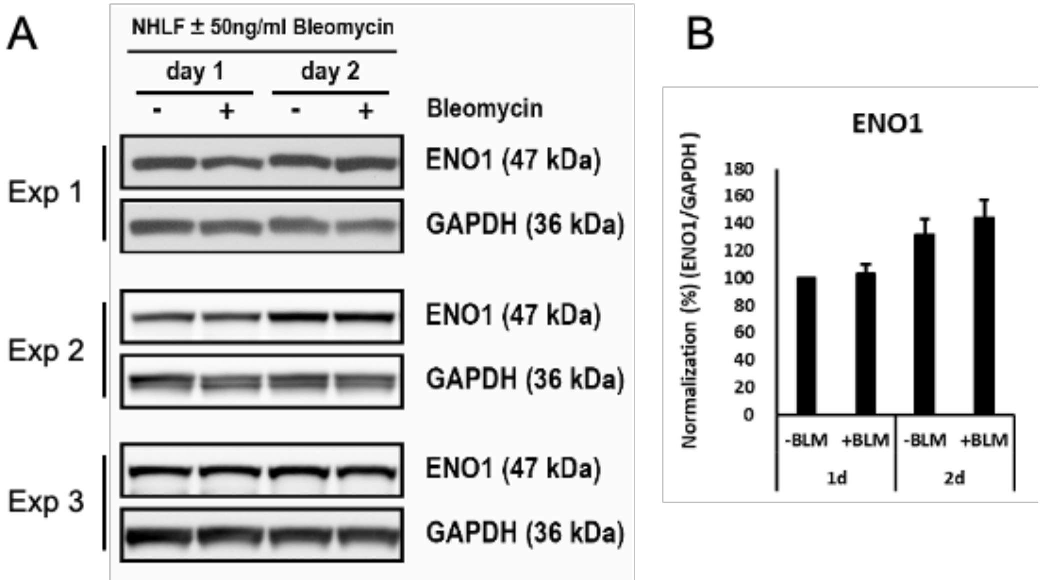

Fig. S5. No significant change of total ENO1 protein expression of NHLF in response to bleomycin treatment.

NHLF cells were treated with 50 ng/ml of bleomycin for 1 or 2 days and protein lysates were separated by 10% SDS-PAGE to determine the expression levels of ENO1. (A) Blots from 3 independent experiments (Exp 1~3) were shown. GAPDH was used as loading control. ENO1 and GAPDH are the same membrane that has undergone two different primary antibody incubations respectively. (B) Densitometry was performed, and quantitative results were shown by % to untreated cells on day 1 (100%).

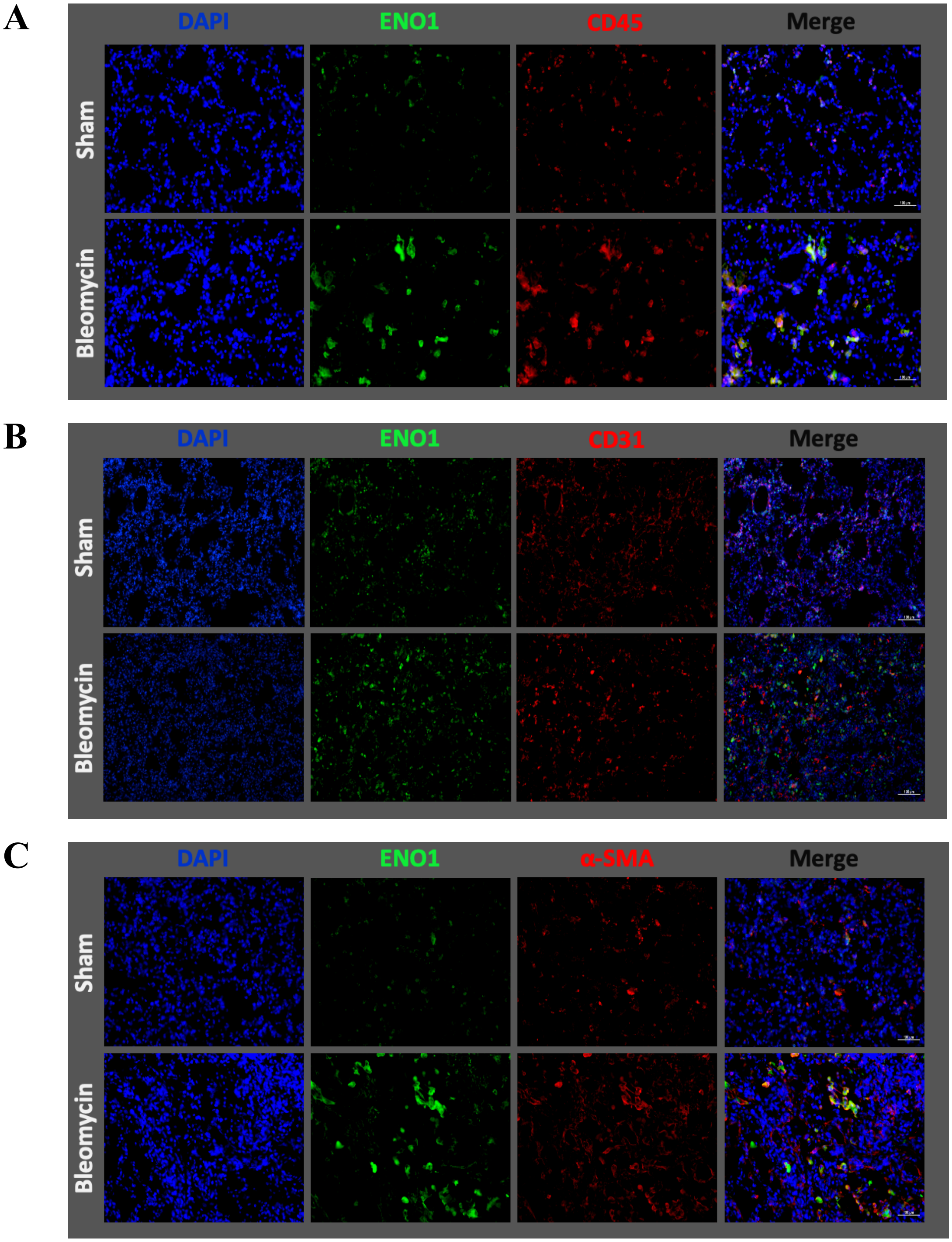

Figure S6. Co-localization ENO1 and CD45 or α-SMA but not CD31.
After intratracheal injection of 3 mg/kg bleomycin (BLM) (day 0), lungs were harvested on day 21. Lung frozen sections were co-stained with ENO1 (green) and CD45 (red) **(A)**, or CD31 (red) **(B)**, or α-SMA (red) **(C)**. Two mice from each group were analyzed. Scale bar: 100 μM

A

B

C

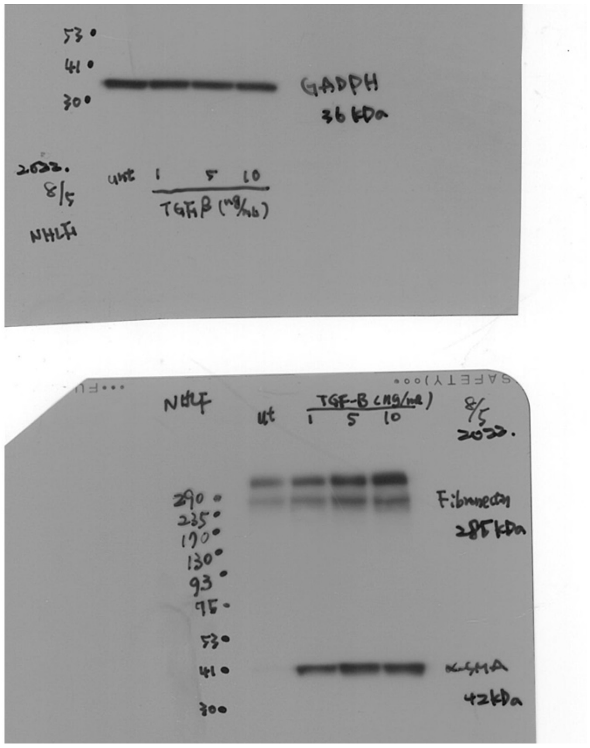

Figure S7. Full length blots of Figure 7B.

Proteins were separated by 10% SDS-PAGE. (A) for GAPDH, (B) for fibronectin (lower band), and (C) for α-SMA. The three proteins are the same membrane that has undergone three different primary antibody incubations respectively.
